# Crystal structure and Hirshfeld surface analysis of 1,2-bis­[5-(4-fluoro­phen­yl)-1,3-oxazol-2-yl]benzene

**DOI:** 10.1107/S2056989026009394

**Published:** 2026-09-11

**Authors:** Viktoriya V. Dyakonenko, Anna M. Shaposhnyk, Rodion Yu. Iliashenko, Andrey O. Doroshenko, Alexander V. Kyrychenko

**Affiliations:** a Institute of Functional Materials Chemistry, SSI "Institute for Single Crystals" of National Academy of Sciences of Ukraine, Nauki Ave 60, Kharkiv 61001, Ukraine; b V. I. Vernadskii Institute of General and Inorganic Chemistry of, National Academy of Sciences of Ukraine, Prospect Palladina 32/34, Kyiv 03680, Ukraine; c V. N. Karazin Kharkiv National University, 4 Svobody Sq., Kharkiv 61022, Ukraine; Universidade de Sâo Paulo, Brazil

**Keywords:** crystal structure, mol­ecular structure, Hirshfeld surface analysis, oxazole

## Abstract

The title compound, C_24_H_14_F_2_N_2_O_2_, was studied by single-crystal and powder X-ray diffraction methods. The title compound crystallizes in the monoclinic space group *P*2_1_/*c* with one mol­ecule in the asymmetric unit. The two chemically equivalent substituents adopt different orientations relative to the central benzene ring. The dihedral angles between the central benzene and oxazole rings are 2.4 (2) and 57.6 (2)°, while those between the oxazole and terminal 4-fluoro­phenyl rings are 13.3 (2) and 2.5 (2)°, respectively. In the crystal, inversion-related mol­ecules form dimers through π–π stacking between oxazole rings [centroid-to-centroid distance = 3.587 (3) Å] and C—H⋯N hydrogen bonds. The dimers are further connected by C—H⋯N inter­actions into layers parallel to (100). Hirshfeld surface analysis indicates that C⋯H/H⋯C (30.2%), H⋯H (21.8%) and F⋯H/H⋯F (18.0%) contacts make the largest contributions to the crystal packing.

## Abstract 2

The title compound crystallizes in the monoclinic space group *P*2_1_/*c* with one mol­ecule in the asymmetric unit. The two chemically equivalent substituents adopt different orientations relative to the central benzene ring. In the crystal, inversion-related mol­ecules form dimers through π–π stacking between oxazole rings and C—H⋯N hydrogen bonds. The dimers are further connected by C—H⋯N inter­actions into layers parallel to (100).

## 1. Chemical context

The title compound belongs to a series of sterically hindered *ortho* analogues of the well-known scintillator dye 1,4-bis­(5-phenyl-1,3-oxazol-2-yl)benzene (POPOP; Ambats & Marsh, 1965 ▸). The approximately planar mol­ecular structure of POPOP promotes extended π-conjugation and efficient fluorescence, accounting for its use in plastic and liquid scintillators (Ott *et al.*, 1957 ▸). In *ortho*-POPOP, however, the two oxazolyl moieties occupy adjacent positions on the central benzene ring. Steric repulsion between these substituents results in pronounced mol­ecular non-planarity both in the crystal and in solution (Doroshenko *et al.*, 1994 ▸, 1997 ▸, 2000*a* ▸, 2002*a* ▸,*b* ▸). The resulting decrease in π-conjugation produces a hypsochromic shift of the absorption spectrum (Doroshenko, 2002*a* ▸). Partial flattening in the electronically excited state restores conjugation and gives rise to bathochromically shifted fluorescence and a large Stokes shift (Doroshenko *et al.*, 1996 ▸, 2000*b* ▸; Doroshenko, 1999 ▸; Iliashenko *et al.*, 2011 ▸).

A characteristic feature of crystalline *ortho*-POPOP derivatives is that the two chemically equivalent oxazolyl substituents may adopt considerably different orientations relative to the central benzene ring (Doroshenko *et al.*, 1994 ▸). The mol­ecular conformation can be described in terms of a relatively planar three-ring benzene–oxazole–phenyl fragment and a two-ring oxazole–phenyl fragment that is strongly inclined to the central benzene ring. A closely related compound is the mono­fluoro-substituted derivative 5-(4-ufloro­phen­yl)-2-[2-(5-phenyl-1,3-oxazol-2-yl)phen­yl]-1,3-oxa­zole (**2**; Ilyashenko *et al.*, 2010 ▸). In its crystal structure, the fluorine atom is disordered between the two terminal phenyl rings, with refined occupancies of 0.627 (3) and 0.373 (3).
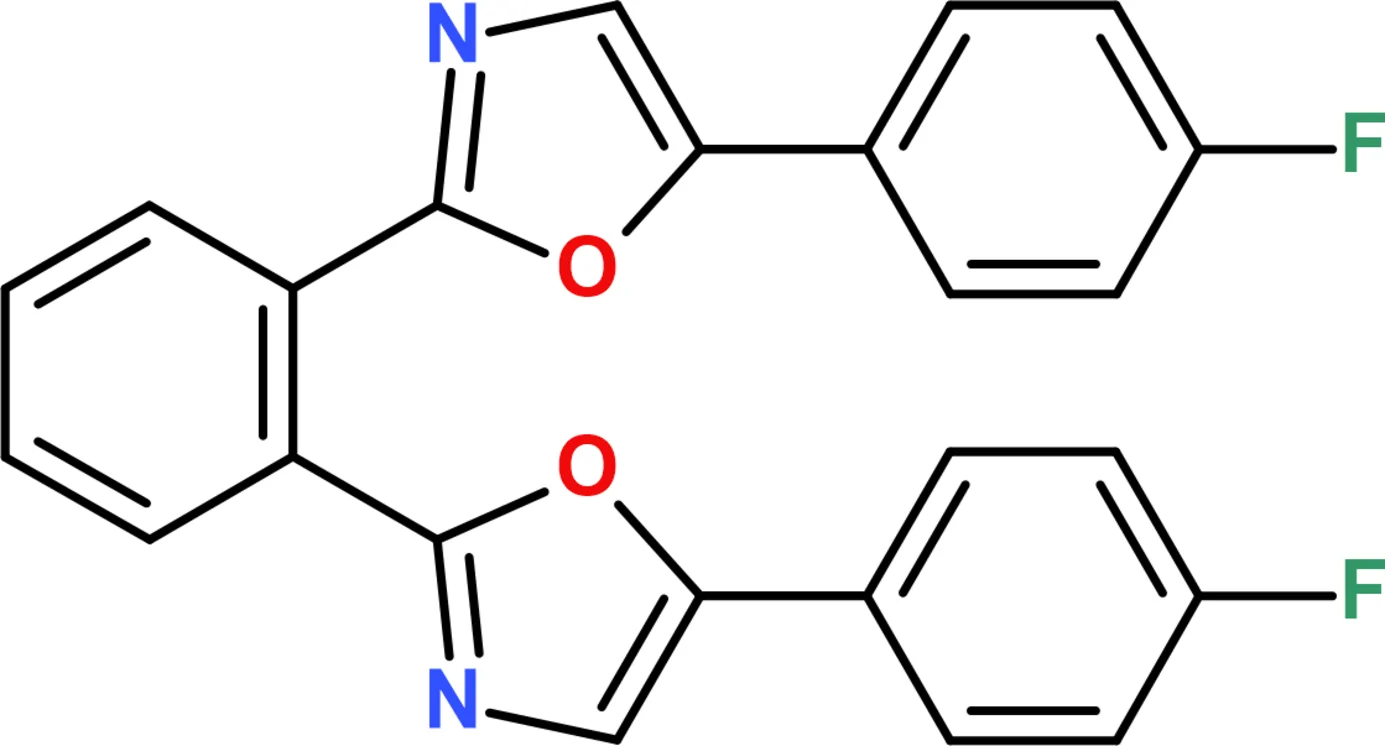


The title compound, 1,2-bis­(5-(4-fluoro­phen­yl)-1,3-oxazol-2-yl)benzene (**1**), is the corresponding di­fluoro-substituted *ortho*-POPOP derivative, in which both terminal phenyl rings bear a *para*-fluoro substituent. The present study reports its mol­ecular and crystal structure and Hirshfeld surface analysis.

## 2. Structural commentary

The mol­ecular structure of the title compond **1** is shown in Fig. 1 ▸. The asymmetric unit contains one crystallographically independent mol­ecule (*Z*′ = 1). The mol­ecule consists of a central benzene ring bearing two 5-(4-fluoro­phen­yl)-1,3-oxazol-2-yl moieties at adjacent *ortho*-positions.

The dihedral angles between the least-squares plane of the central benzene ring (C10–C15) and those of the oxazole rings O1/N1/C7–C9 and O2/N2/C16–C18 are 2.4 (2) and 57.6 (2)°, respectively. The corresponding dihedral angles between the oxazole rings and the terminal 4-fluoro­phenyl rings C1–C6 and C19–C24 are 13.4 (2) and 2.5 (2)°, respectively. Consequently, one moiety forms an approximately planar benzene–oxazole–4-fluoro­phenyl fragment, with only a slight rotation of the terminal 4-fluoro­phenyl ring. In the second moiety, the oxazole and 4-fluoro­phenyl rings are essentially coplanar, whereas their mean plane is strongly inclined to that of the central benzene ring. Thus, although the two substituents are chemically equivalent, they adopt different orientations relative to the central benzene ring in the crystal. Similar differences in the orientations of chemically equivalent substituents have been observed in other crystalline *ortho*-POPOP derivatives (Doroshenko *et al.*, 1994 ▸, 1997 ▸, 2000*a* ▸, 2002*b* ▸).

## 3. Supra­molecular features

In the crystal, inversion-related mol­ecules form dimers consolidated π–π stacking inter­actions between the O1/N1/C7–C9 oxazole rings (Fig. 2 ▸). The centroid-to-centroid distance *Cg*1⋯*Cg*1^iv^ is 3.587 (3) Å, with a perpendicular distance of 3.456 Å and a shift of 0.962 Å [*Cg*1 is the centroid of the O1/N1/C7–C9 ring; symmetry code: (iv) 1 − *x*, 1 − *y*, 1 – *z*]. The dimers are additionally consolidated by pairs of C24—H24⋯N1^iv^ hydrogen bonds (Table 1 ▸).

The inversion-related dimers are connected by C8—H8⋯N2^ii^ hydrogen bonds, forming chains extending parallel to [010]. Together, the C—H⋯N hydrogen bonds and π–π inter­actions generate layers parallel to (100), as shown in Fig. 3 ▸. The terminal fluorine atoms are directed towards the inter­layer region. Weak C5—H5⋯F2^i^ contacts are observed between adjacent layers along the *a*-axis direction (Table 1 ▸) and may contribute to the consolidation of the crystal packing (Thalladi *et al.*, 1998 ▸). A further weak C23—H23⋯F1^iii^ contact occurs within the layers (Table 1 ▸).

## 4. Powder X-ray diffraction

The powder diffraction pattern of **1** was recorded using a Rigaku SmartLab powder diffractometer at room temperature (Cu *K*α radiation, Bragg–Brentano geometry, θ–θ scanning, Ni filter, 3° < 2θ < 60°, Δ2θ = 0.01°). The Rietveld refinement of the obtained pattern (Fig. 4 ▸) was carried out using the *FullProf* and *WinPLOTR* programs (Rodríguez-Carvajal & Roisnel, 1998 ▸), with data from the external NIST SRM 640 standard used for the calculation of the instrumental profile function and the single-crystal structure used as the structural model for refinement. A minor preferred orientation effect along the [110] direction was revealed during the refinement and taken into account. The crystallite size obtained from the refinement was approximately 99 nm, indicating relatively large crystallites and no pronounced nanocrystalline character of the sample. The main results of the Rietveld refinement are summarized in Table 2 ▸. The experimental powder X-ray diffraction pattern is in good agreement with the theoretical pattern calculated from the single-crystal structure data.

## 5. Hirshfeld surface analysis and fingerprint plots

A Hirshfeld surface analysis was performed using *CrystalExplorer21* (Spackman *et al.*, 2021 ▸) to examine the inter­molecular contacts in the crystal of **1**. The Hirshfeld surface mapped over *d*_norm_ (Spackman & Jayatilaka, 2009 ▸) is shown in Fig. 5 ▸, while the corresponding two-dimensional fingerprint plots (McKinnon *et al.*, 2007 ▸) are presented in Fig. 6 ▸. The red spots on the *d*_norm_ surface correspond to the C—H⋯N contacts described above.

The decomposed fingerprint plots show that C⋯H/H⋯C contacts make the largest contribution to the Hirshfeld surface, accounting for 30.2%, followed by H⋯H contacts (21.8%) and F⋯H/H⋯F contacts (18.0%). The relatively high contribution of F⋯H/H⋯F contacts reflects the frequent close approaches between the fluorine atoms and hydrogen atoms of neighbouring mol­ecules. These include the weak inter­layer C5—H5⋯F2^i^ and intra­layer C23—H23⋯F1^iii^ contacts described above. Thus, contacts involving fluorine atoms contribute to the mol­ecular association both within and between the layers.

The N⋯H/H⋯N contacts account for 8.1% of the Hirshfeld surface and are consistent with the C—H⋯N inter­actions described in the *Supra­molecular features* section. The C⋯C contacts contribute 5.4% to the Hirshfeld surface and support the presence of π–π stacking inter­actions between inversion-related oxazole rings.

Smaller contributions arise from O⋯H/H⋯O and O⋯N/N⋯O contacts, each accounting for 2.9%, as well as from N⋯C/C⋯N (1.5%), F⋯F (1.4%), O⋯C/C⋯O (1.0%) and F⋯C/C⋯F (0.8%) contacts. These minor contributions represent other weak contacts present in the crystal packing.

## 6. Database survey

An expanded substructure search of the Cambridge Structural Database (CSD, Version 6.01, updated February 2026; Groom *et al.*, 2016 ▸), based on the ortho-disubstituted central benzene core and related five-membered *O*,*N*-heterocycles, gave ten entries. Three hits corresponding to bis­(imidazole) derivatives (GUVNET, GUVNET01 and SODQEJ) were excluded because they do not contain oxazole or oxa­diazole rings. The remaining seven entries correspond to six unique related crystal structures, since PODZEN and PODZEN10 describe the same crystallographic model.

The closest bis­(1,3-oxazole) analogues are the unsubstituted ortho-POPOP mol­ecule (PODZEN/PODZEN10; Doroshenko *et al.*, 1994 ▸) and its mono­fluoro derivative (SUZVEP; Ilyashenko *et al.*, 2010 ▸). Three unsymmetrical analogues containing one 1,3-oxazole and one 1,3,4-oxa­diazole ring were also found: NODYOU (Doroshenko *et al.*, 1997 ▸), EDEFIC (Doroshenko *et al.*, 2000*a* ▸) and HUGJUO (Doroshenko *et al.*, 2002*b* ▸). The more distant bis­(1,3,4-oxa­diazole) analogue SEQREN was reported by Yang *et al.* (2010 ▸).

All these structures adopt non-planar mol­ecular geometries in which the two substituents have different orientations relative to the central benzene ring. In PODZEN/PODZEN10, the dihedral angles between the plane of the central benzene ring and the planes of the two oxazole rings are 10.3 and 66.9°, respectively. The corresponding published values for SUZVEP are 10.7 and 64.1° (Ilyashenko *et al.*, 2010 ▸). In the title compound, these angles are 2.37 and 57.60°, indicating somewhat smaller twisting of both oxazole rings relative to the central benzene ring, although the difference between the orientations of the two oxazole moieties, characteristic of the *ortho*-POPOP framework, is retained.

## 7. Synthesis and crystallization

The title compound **1** was synthesized by the reported procedure (Doroshenko *et al.*, 1994 ▸) using phthaloyl dichloride and 4-F-substituted ω-amino-aceto­phenone hydro­chloride (Ilyashenko *et al.*, 2010 ▸). A solution of phthaloyl dichloride (0.025 mol, ∼3.6 mL) in 20 mL of benzene was mixed with 9.5 g (0.05 mol) 4-F-ω-amino­aceto­phenone hydro­chloride in 30 mL of water. The reaction mixture was basified dropwise with concentrated aqueous sodium carbonate with continuous stirring to keep it weakly alkaline and the mixture was stirred for 1 h. Then the resulting precipitate was filtered off, washed with water and air-dried at room temperature. The obtained inter­mediate was dissolved in 80 ml of concentrated sulfuric acid, stirred 30 min at room temperature, and then for an additional 30 min at 328 K. The reaction mixture was cooled, poured onto ice, the product was filtered off, thoroughly washed with water until the washings were neutral, air-dried and finally recrystallized from ethanol. Yield: 5.9 g (58%); colorless crystals.

Crystals suitable for X-ray diffraction analysis were obtained by slow evaporation of a concentrated solution of **1** in propan-2-ol.

## 8. Refinement

Crystal data, data collection and structure refinement details are summarized in Table 3 ▸. The H atoms were placed in calculated positions (C—H = 0.93 Å) and refined using a riding model with *U*_iso_(H) = 1.2*U*_eq_ of the carrier atom.

## Supplementary Material

Crystal structure: contains datablock(s) I. DOI: 10.1107/S2056989026009394/ex2105sup1.cif

Structure factors: contains datablock(s) I. DOI: 10.1107/S2056989026009394/ex2105Isup2.hkl

Supporting information file. DOI: 10.1107/S2056989026009394/ex2105Isup3.cml

CCDC reference: 2586503

Additional supporting information:  crystallographic information; 3D view; checkCIF report

## Figures and Tables

**Figure 1:**
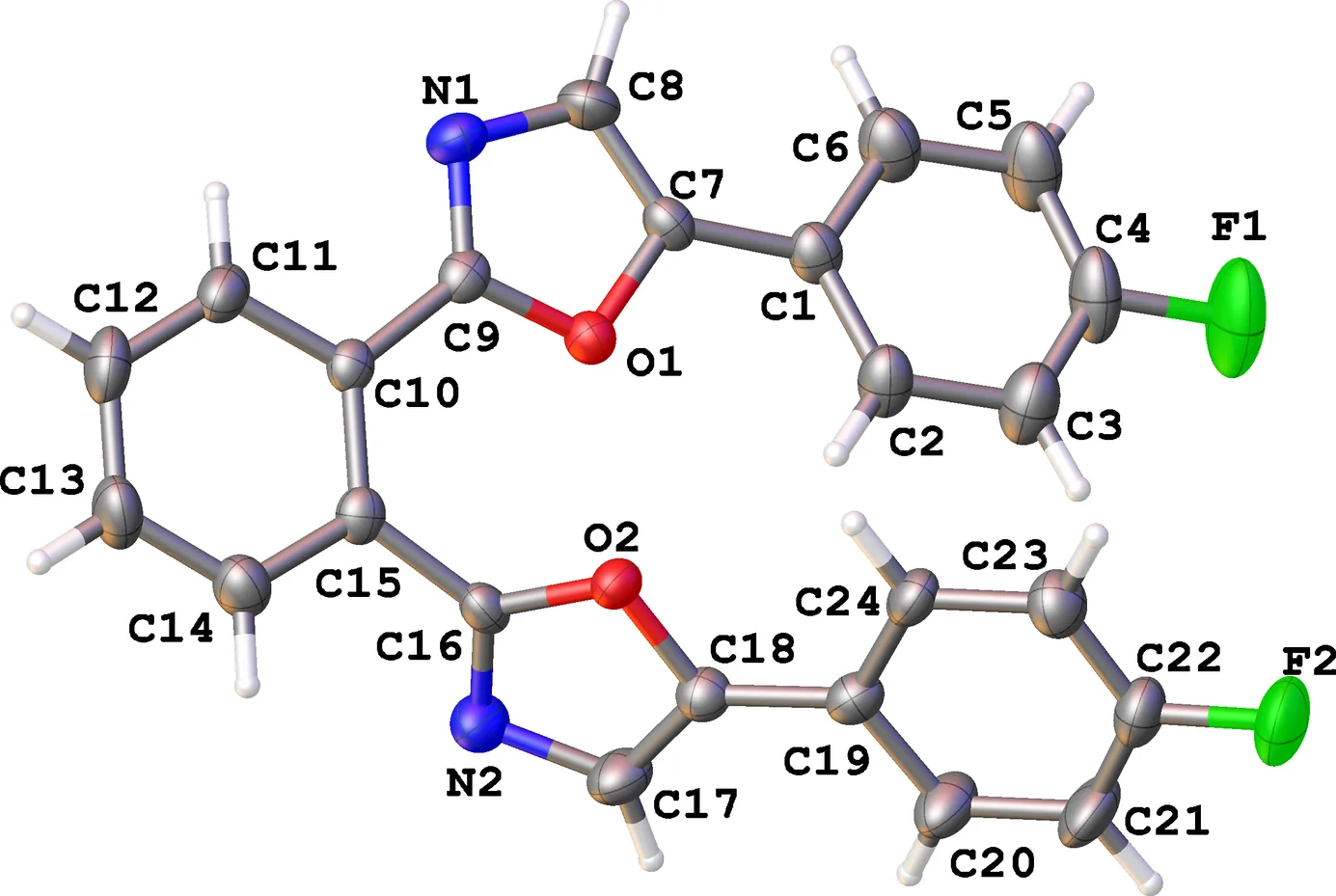
The mol­ecular structure of **1**, showing the atom labelling and displacement ellipsoids drawn at the 50% probability level.

**Figure 2:**
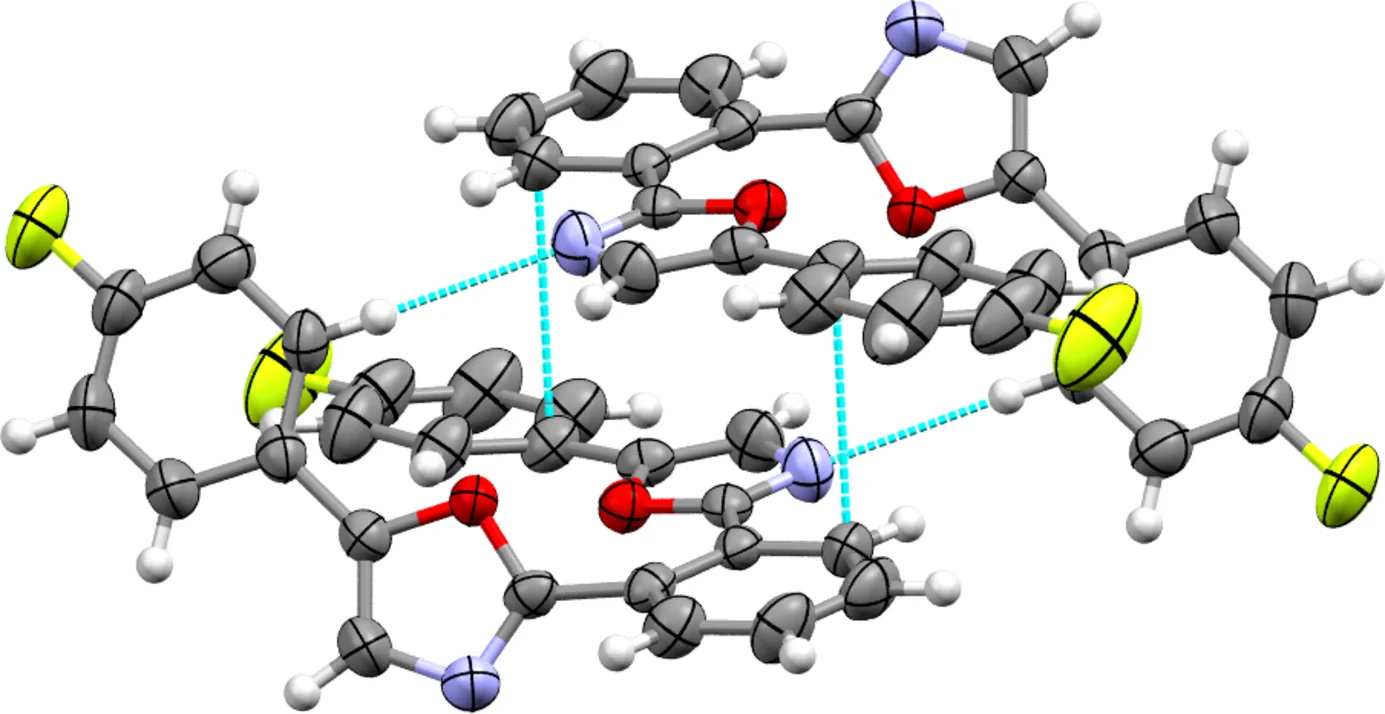
A centrosymmetric dimer of **1** formed by π–π stacking and C—H⋯N hydrogen bonds. The inter­molecular inter­actions are shown as dashed lines. [Symmetry code: (iv) 1 − x, 1 − y, 1 − z.]

**Figure 3:**
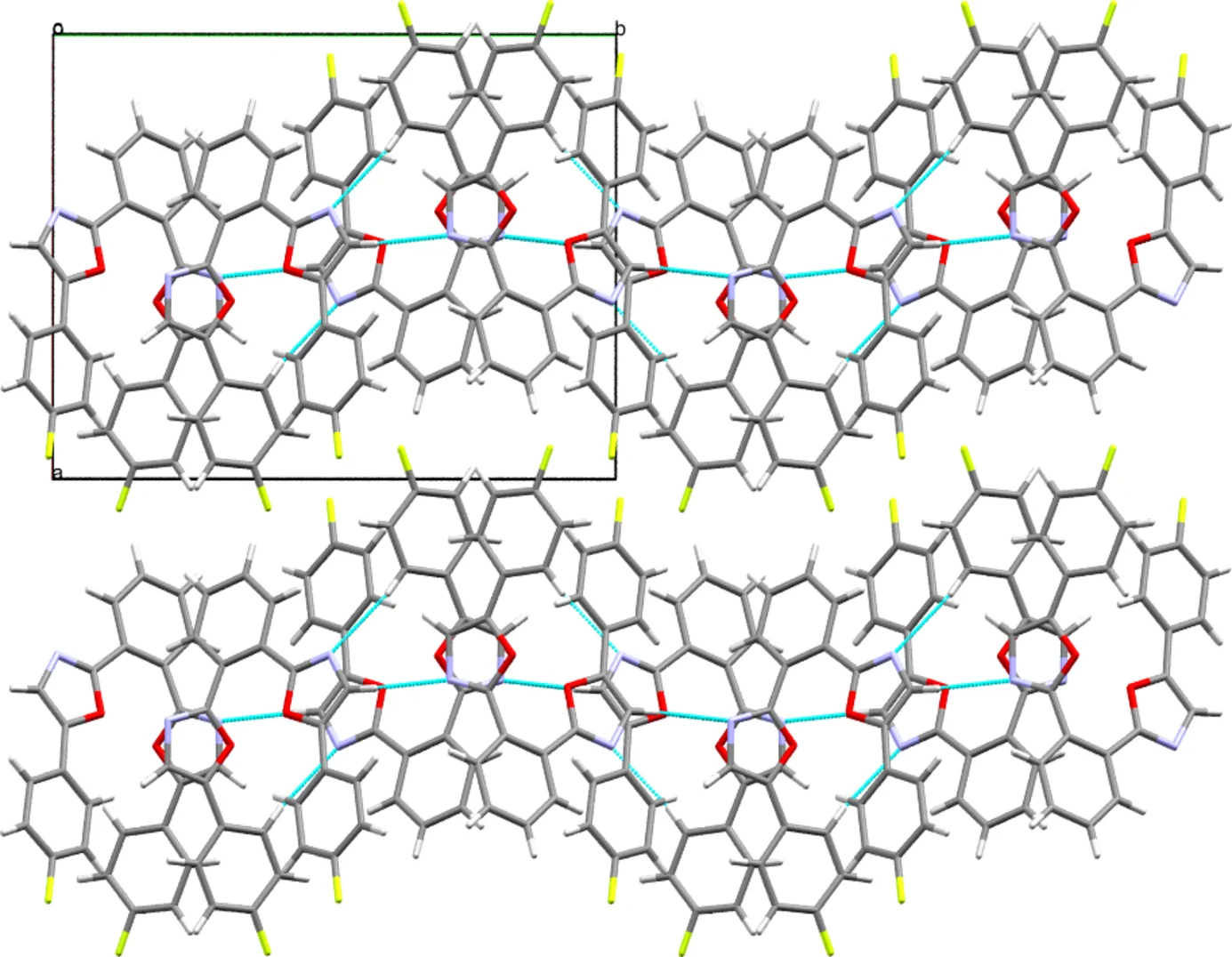
Crystal packing of **1** showing the C—H⋯N hydrogen-bonded layers parallel to (100).

**Figure 4:**
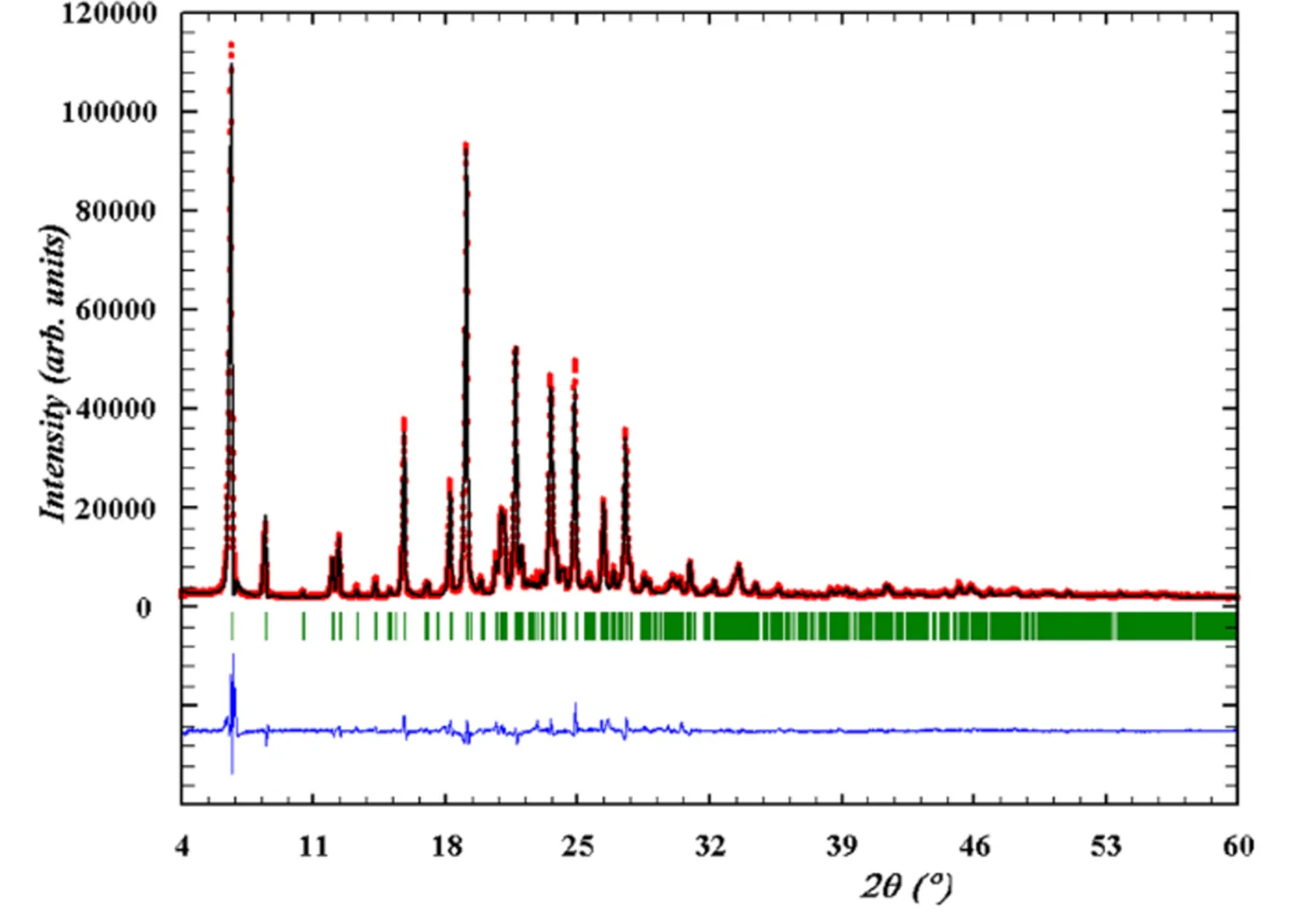
Final Rietveld plot of compound **1**. Observed data points are indicated by red dots, the best-fit profile (upper trace) and the difference pattern (lower trace) are shown as solid lines. The vertical bars correspond to the positions of the Bragg peaks.

**Figure 5:**
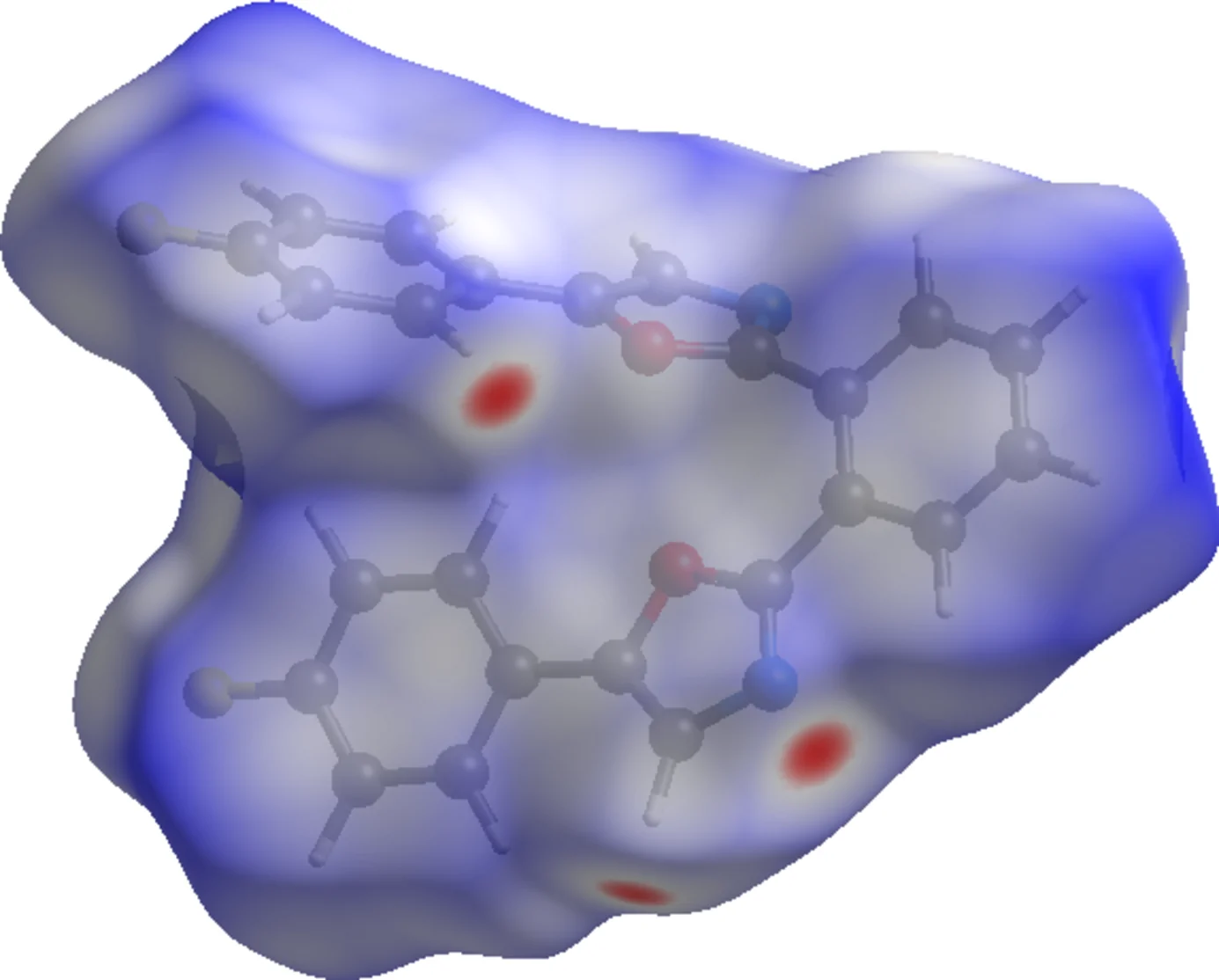
Three-dimensional Hirshfeld surface of compound 1 mapped over d_norm_.

**Figure 6:**
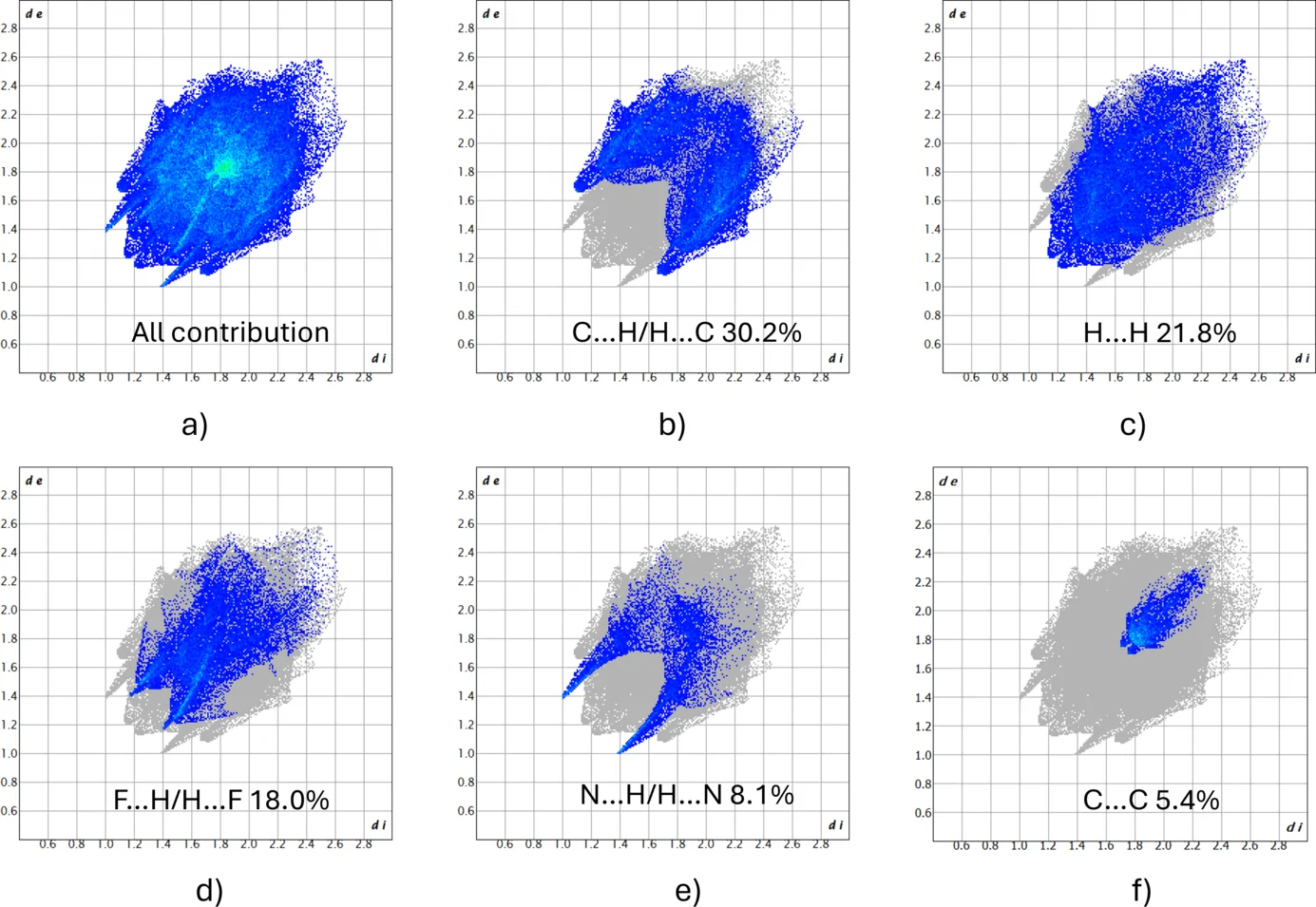
Two-dimensional fingerprint plots for compound **1** showing (a) all inter­molecular contacts and the plots delineated into contributions from (b) C⋯H/H⋯C, (c) H⋯H, (d) F⋯H/H⋯F, (e) N⋯H/H⋯N, (f) C⋯C contacts. The qu­anti­ties d_e_ and d_i_ represent the distances from a point on the Hirshfeld surface to the nearest atom outside and inside the surface, respectively.

**Table 1:** Hydrogen-bond geometry (Å, °)

*D*—H⋯*A*	*D*—H	H⋯*A*	*D*⋯*A*	*D*—H⋯*A*
C5—H5⋯F2^i^	0.93	2.70	3.470 (5)	141
C8—H8⋯N2^ii^	0.93	2.56	3.489 (6)	176
C23—H23⋯F1^iii^	0.93	2.82	3.559 (5)	137
C24—H24⋯N1^iv^	0.93	2.54	3.449 (5)	168

Symmetry codes: (i) 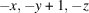; (ii) 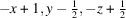; (iii) 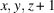; (iv) 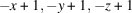.

**Table 2:** Experimental data of the X-ray powder diffraction study performed at 293 K

Crystal system, space group	Monoclinic, *P*2_1_/*c*
*a* (Å)	13.7470 (5)
*b* (Å)	16.9219 (4)
*c* (Å)	8.4473 (1)
β (°)	104.432 (2)
*V* (Å^3^)	1903.04 (8)
*D*_*x*_, Mg m^−3^	1.397
Apparent crystallite size (nm)	99
	
Refinement	
*R*_p_	0.0590
*R*_wp_	0.0942
*R*_exp _	0.0147
*R*_b_	0.0592
*R*_f_	0.0561

**Table 3:** Experimental details

Crystal data	
Chemical formula	C_24_H_14_F_2_N_2_O_2_
*M*_r_	400.37
Crystal system, space group	Monoclinic, *P*2_1_/*c*
Temperature (K)	296
*a*, *b*, *c* (Å)	13.767 (8), 16.914 (11), 8.447 (5)
β (°)	104.423 (13)
*V* (Å^3^)	1904.9 (19)
*Z*	4
Radiation type	Mo *K*α
μ (mm^−1^)	0.10
Crystal size (mm)	0.3 × 0.2 × 0.1

Data collection	
Diffractometer	Bruker APEXII CCD
Absorption correction	Multi-scan (*SADABS*; Krause *et al.*, 2015 ▸)
*T*_min_, *T*_max_	0.617, 0.746
No. of measured, independent and observed [*I* > 2σ(*I*)] reflections	10872, 3740, 1446
*R*_int_	0.110
(sin θ/λ)_max_ (Å^−1^)	0.617

Refinement	
*R*[*F*^2^ > 2σ(*F*^2^)], *wR*(*F*^2^), *S*	0.076, 0.151, 0.91
No. of reflections	3740
No. of parameters	272
H-atom treatment	H-atom parameters constrained
Δρ_max_, Δρ_min_ (e Å^−3^)	0.20, −0.19

Computer programs: *APEX2* and *SAINT* (Bruker, 2008 ▸), *SHELXT2018/2* (Sheldrick, 2015*a* ▸), *SHELXL2019/3* (Sheldrick, 2015*b* ▸) and *OLEX2* (Dolomanov *et al.*, 2009 ▸).

## supplementary crystallographic information

### 1,2-Bis[5-(4-fluorophenyl)-1,3-oxazol-2-yl]benzene Crystal data

**Table:**

C_24_H_14_F_2_N_2_O_2_	*F*(000) = 824
*M**_r_* = 400.37	*D*_x_ = 1.396 Mg m^−^^3^
Monoclinic, *P*2_1_/*c*	Mo *K*α radiation, λ = 0.71073 Å
*a* = 13.767 (8) Å	Cell parameters from 633 reflections
*b* = 16.914 (11) Å	θ = 2.4–22.8°
*c* = 8.447 (5) Å	µ = 0.10 mm^−^^1^
β = 104.423 (13)°	*T* = 296 K
*V* = 1904.9 (19) Å^3^	Block, colourless
*Z* = 4	0.3 × 0.2 × 0.1 mm

### 1,2-Bis[5-(4-fluorophenyl)-1,3-oxazol-2-yl]benzene Data collection

**Table:**

Bruker APEXII CCD diffractometer	1446 reflections with *I* > 2σ(*I*)
φ and ω scans	*R*_int_ = 0.110
Absorption correction: multi-scan (SADABS; Krause *et al.*, 2015)	θ_max_ = 26.0°, θ_min_ = 1.5°
*T*_min_ = 0.617, *T*_max_ = 0.746	*h* = −16→16
10872 measured reflections	*k* = −20→20
3740 independent reflections	*l* = −8→10

### 1,2-Bis[5-(4-fluorophenyl)-1,3-oxazol-2-yl]benzene Refinement

**Table:**

Refinement on *F*^2^	Hydrogen site location: inferred from neighbouring sites
Least-squares matrix: full	H-atom parameters constrained
*R*[*F*^2^ > 2σ(*F*^2^)] = 0.076	*w* = 1/[σ^2^(*F*_o_^2^) + (0.045*P*)^2^] where *P* = (*F*_o_^2^ + 2*F*_c_^2^)/3
*wR*(*F*^2^) = 0.151	(Δ/σ)_max_ < 0.001
*S* = 0.91	Δρ_max_ = 0.20 e Å^−^^3^
3740 reflections	Δρ_min_ = −0.19 e Å^−^^3^
272 parameters	Extinction correction: *SHELXL2019/3* (Sheldrick, 2015b), Fc^*^=kFc[1+0.001xFc^2^λ^3^/sin(2θ)]^-1/4^
0 restraints	Extinction coefficient: 0.0043 (8)

### 1,2-Bis[5-(4-fluorophenyl)-1,3-oxazol-2-yl]benzene Special details

**Table:**

Geometry. All esds (except the esd in the dihedral angle between two l.s. planes) are estimated using the full covariance matrix. The cell esds are taken into account individually in the estimation of esds in distances, angles and torsion angles; correlations between esds in cell parameters are only used when they are defined by crystal symmetry. An approximate (isotropic) treatment of cell esds is used for estimating esds involving l.s. planes.
Refinement. Using Olex2 (Dolomanov *et al.*, 2009), the structure was solved with the SHELXT (Sheldrick, 2015*a*) structure solution program using Intrinsic Phasing and refined with the *SHELXL* (Sheldrick, 2015*b*) refinement package. Full-matrix least squares refinement against F^2^ in anisotropic approximation was used for non-hydrogen atoms.

### 1,2-Bis[5-(4-fluorophenyl)-1,3-oxazol-2-yl]benzene Fractional atomic coordinates and isotropic or equivalent isotropic displacement parameters (Å^2^)

**Table:**

	*x*	*y*	*z*	*U*_iso_*/*U*_eq_	
F1	0.0504 (2)	0.4923 (2)	−0.1473 (3)	0.1238 (14)	
F2	−0.06623 (16)	0.61801 (17)	0.4548 (3)	0.0916 (10)	
O1	0.47053 (18)	0.58652 (15)	0.3229 (3)	0.0414 (7)	
O2	0.39878 (17)	0.68355 (14)	0.5272 (3)	0.0409 (7)	
N1	0.6035 (2)	0.5080 (2)	0.3606 (4)	0.0538 (10)	
N2	0.4544 (2)	0.7954 (2)	0.4465 (4)	0.0585 (10)	
C1	0.3414 (3)	0.5178 (3)	0.1210 (5)	0.0474 (11)	
C2	0.2666 (3)	0.5654 (3)	0.1507 (5)	0.0583 (13)	
H2	0.282616	0.603993	0.231339	0.070*	
C3	0.1670 (3)	0.5564 (3)	0.0611 (6)	0.0762 (16)	
H3	0.115874	0.587502	0.082073	0.091*	
C4	0.1481 (4)	0.5002 (4)	−0.0581 (6)	0.0791 (17)	
C5	0.2189 (4)	0.4515 (3)	−0.0906 (6)	0.0819 (18)	
H5	0.202142	0.413168	−0.171779	0.098*	
C6	0.3169 (3)	0.4603 (3)	0.0002 (5)	0.0675 (14)	
H6	0.366723	0.427564	−0.019925	0.081*	
C7	0.4445 (3)	0.5227 (2)	0.2193 (5)	0.0399 (10)	
C8	0.5244 (3)	0.4759 (2)	0.2423 (5)	0.0551 (12)	
H8	0.526946	0.428583	0.187402	0.066*	
C9	0.5673 (3)	0.5719 (2)	0.4056 (5)	0.0386 (10)	
C10	0.6219 (3)	0.6282 (2)	0.5302 (4)	0.0360 (10)	
C11	0.7223 (3)	0.6099 (2)	0.6003 (5)	0.0517 (12)	
H11	0.749581	0.564028	0.568470	0.062*	
C12	0.7819 (3)	0.6586 (3)	0.7162 (5)	0.0623 (14)	
H12	0.848343	0.645215	0.763227	0.075*	
C13	0.7421 (3)	0.7271 (3)	0.7613 (5)	0.0643 (14)	
H13	0.782182	0.760607	0.837773	0.077*	
C14	0.6433 (3)	0.7465 (3)	0.6938 (5)	0.0555 (12)	
H14	0.617286	0.792681	0.726634	0.067*	
C15	0.5814 (3)	0.6980 (2)	0.5770 (5)	0.0392 (10)	
C16	0.4786 (3)	0.7270 (2)	0.5112 (5)	0.0400 (10)	
C17	0.3507 (3)	0.7967 (3)	0.4137 (5)	0.0591 (13)	
H17	0.310674	0.838583	0.364357	0.071*	
C18	0.3158 (3)	0.7298 (2)	0.4625 (5)	0.0438 (11)	
C19	0.2165 (3)	0.6990 (2)	0.4603 (5)	0.0447 (11)	
C20	0.1319 (3)	0.7432 (3)	0.3893 (6)	0.0688 (14)	
H20	0.139112	0.792099	0.343075	0.083*	
C21	0.0372 (3)	0.7154 (3)	0.3868 (6)	0.0789 (16)	
H21	−0.019404	0.744990	0.339041	0.095*	
C22	0.0282 (3)	0.6444 (3)	0.4549 (6)	0.0614 (13)	
C23	0.1077 (3)	0.5980 (3)	0.5230 (5)	0.0606 (13)	
H23	0.098947	0.549031	0.567457	0.073*	
C24	0.2030 (3)	0.6262 (3)	0.5245 (5)	0.0501 (11)	
H24	0.258774	0.595131	0.569852	0.060*	

### 1,2-Bis[5-(4-fluorophenyl)-1,3-oxazol-2-yl]benzene Atomic displacement parameters (Å^2^)

**Table:**

	*U*^11^	*U*^22^	*U*^33^	*U*^12^	*U*^13^	*U*^23^
F1	0.0618 (19)	0.213 (4)	0.087 (2)	−0.037 (2)	0.0004 (17)	−0.033 (2)
F2	0.0395 (15)	0.121 (3)	0.114 (2)	−0.0090 (16)	0.0200 (15)	−0.004 (2)
O1	0.0399 (16)	0.0409 (17)	0.0429 (17)	−0.0019 (14)	0.0093 (13)	−0.0034 (14)
O2	0.0350 (15)	0.0380 (16)	0.0513 (18)	0.0020 (14)	0.0137 (13)	0.0023 (14)
N1	0.048 (2)	0.048 (2)	0.065 (3)	0.0102 (19)	0.014 (2)	−0.004 (2)
N2	0.050 (2)	0.047 (2)	0.082 (3)	−0.0011 (19)	0.021 (2)	0.007 (2)
C1	0.045 (3)	0.055 (3)	0.045 (3)	−0.008 (2)	0.016 (2)	−0.006 (2)
C2	0.046 (3)	0.080 (4)	0.049 (3)	−0.009 (3)	0.013 (2)	−0.012 (3)
C3	0.050 (3)	0.106 (5)	0.072 (4)	−0.003 (3)	0.013 (3)	−0.006 (3)
C4	0.052 (4)	0.128 (6)	0.052 (3)	−0.031 (3)	0.005 (3)	−0.012 (3)
C5	0.066 (4)	0.121 (5)	0.058 (4)	−0.026 (4)	0.016 (3)	−0.032 (3)
C6	0.063 (3)	0.080 (4)	0.061 (3)	−0.014 (3)	0.017 (3)	−0.016 (3)
C7	0.046 (3)	0.038 (3)	0.036 (3)	−0.008 (2)	0.011 (2)	−0.005 (2)
C8	0.059 (3)	0.042 (3)	0.066 (3)	0.001 (3)	0.017 (3)	−0.014 (2)
C9	0.037 (2)	0.040 (3)	0.039 (3)	0.001 (2)	0.012 (2)	0.010 (2)
C10	0.033 (2)	0.040 (2)	0.036 (2)	−0.002 (2)	0.0108 (19)	0.006 (2)
C11	0.037 (2)	0.050 (3)	0.069 (3)	0.002 (2)	0.015 (2)	0.008 (3)
C12	0.034 (3)	0.083 (4)	0.064 (3)	−0.012 (3)	0.002 (2)	0.008 (3)
C13	0.047 (3)	0.076 (4)	0.067 (3)	−0.018 (3)	0.009 (2)	−0.020 (3)
C14	0.052 (3)	0.058 (3)	0.057 (3)	−0.007 (2)	0.014 (2)	−0.011 (2)
C15	0.036 (2)	0.047 (3)	0.036 (2)	−0.003 (2)	0.012 (2)	0.003 (2)
C16	0.043 (3)	0.034 (3)	0.046 (3)	−0.008 (2)	0.016 (2)	−0.007 (2)
C17	0.053 (3)	0.049 (3)	0.076 (3)	0.013 (2)	0.018 (3)	0.014 (3)
C18	0.043 (3)	0.044 (3)	0.044 (3)	0.002 (2)	0.010 (2)	0.005 (2)
C19	0.043 (3)	0.046 (3)	0.045 (3)	0.008 (2)	0.012 (2)	0.002 (2)
C20	0.048 (3)	0.062 (3)	0.096 (4)	0.011 (3)	0.017 (3)	0.026 (3)
C21	0.037 (3)	0.087 (4)	0.110 (4)	0.019 (3)	0.014 (3)	0.017 (4)
C22	0.036 (3)	0.078 (4)	0.071 (4)	−0.002 (3)	0.013 (2)	−0.002 (3)
C23	0.054 (3)	0.063 (3)	0.066 (3)	−0.007 (3)	0.016 (3)	0.004 (3)
C24	0.032 (2)	0.058 (3)	0.057 (3)	0.008 (2)	0.005 (2)	−0.001 (2)

### 1,2-Bis[5-(4-fluorophenyl)-1,3-oxazol-2-yl]benzene Geometric parameters (Å, º)

**Table:**

F1—C4	1.374 (5)	C10—C11	1.396 (4)
F2—C22	1.375 (5)	C10—C15	1.404 (5)
O1—C7	1.379 (4)	C11—H11	0.9300
O1—C9	1.365 (4)	C11—C12	1.382 (5)
O2—C16	1.356 (4)	C12—H12	0.9300
O2—C18	1.380 (4)	C12—C13	1.376 (6)
N1—C8	1.392 (5)	C13—H13	0.9300
N1—C9	1.286 (4)	C13—C14	1.377 (5)
N2—C16	1.288 (5)	C14—H14	0.9300
N2—C17	1.386 (5)	C14—C15	1.397 (5)
C1—C2	1.378 (5)	C15—C16	1.470 (5)
C1—C6	1.388 (5)	C17—H17	0.9300
C1—C7	1.458 (5)	C17—C18	1.334 (5)
C2—H2	0.9300	C18—C19	1.459 (5)
C2—C3	1.401 (5)	C19—C20	1.388 (5)
C3—H3	0.9300	C19—C24	1.376 (5)
C3—C4	1.362 (6)	C20—H20	0.9300
C4—C5	1.356 (6)	C20—C21	1.382 (6)
C5—H5	0.9300	C21—H21	0.9300
C5—C6	1.383 (5)	C21—C22	1.349 (6)
C6—H6	0.9300	C22—C23	1.352 (5)
C7—C8	1.329 (5)	C23—H23	0.9300
C8—H8	0.9300	C23—C24	1.394 (5)
C9—C10	1.479 (5)	C24—H24	0.9300
			
C9—O1—C7	104.4 (3)	C13—C12—C11	119.4 (4)
C16—O2—C18	105.4 (3)	C13—C12—H12	120.3
C9—N1—C8	104.4 (3)	C12—C13—H13	119.8
C16—N2—C17	104.0 (3)	C12—C13—C14	120.4 (4)
C2—C1—C6	119.2 (4)	C14—C13—H13	119.8
C2—C1—C7	121.9 (4)	C13—C14—H14	119.4
C6—C1—C7	118.9 (4)	C13—C14—C15	121.2 (4)
C1—C2—H2	119.5	C15—C14—H14	119.4
C1—C2—C3	120.9 (4)	C10—C15—C16	125.9 (3)
C3—C2—H2	119.5	C14—C15—C10	118.5 (4)
C2—C3—H3	121.4	C14—C15—C16	115.6 (4)
C4—C3—C2	117.1 (5)	O2—C16—C15	120.6 (3)
C4—C3—H3	121.4	N2—C16—O2	113.6 (3)
C3—C4—F1	117.0 (5)	N2—C16—C15	125.6 (4)
C5—C4—F1	119.0 (5)	N2—C17—H17	124.5
C5—C4—C3	124.0 (5)	C18—C17—N2	111.0 (4)
C4—C5—H5	120.9	C18—C17—H17	124.5
C4—C5—C6	118.2 (5)	O2—C18—C19	118.9 (4)
C6—C5—H5	120.9	C17—C18—O2	106.1 (4)
C1—C6—H6	119.7	C17—C18—C19	135.0 (4)
C5—C6—C1	120.5 (5)	C20—C19—C18	119.7 (4)
C5—C6—H6	119.7	C24—C19—C18	122.1 (4)
O1—C7—C1	117.9 (4)	C24—C19—C20	118.2 (4)
C8—C7—O1	107.4 (3)	C19—C20—H20	119.7
C8—C7—C1	134.5 (4)	C21—C20—C19	120.6 (4)
N1—C8—H8	125.0	C21—C20—H20	119.7
C7—C8—N1	110.1 (4)	C20—C21—H21	120.6
C7—C8—H8	125.0	C22—C21—C20	118.9 (4)
O1—C9—C10	120.4 (3)	C22—C21—H21	120.6
N1—C9—O1	113.7 (3)	C21—C22—F2	118.4 (4)
N1—C9—C10	125.9 (4)	C21—C22—C23	123.2 (4)
C11—C10—C9	115.6 (4)	C23—C22—F2	118.4 (5)
C11—C10—C15	119.2 (4)	C22—C23—H23	121.1
C15—C10—C9	125.1 (3)	C22—C23—C24	117.8 (4)
C10—C11—H11	119.4	C24—C23—H23	121.1
C12—C11—C10	121.2 (4)	C19—C24—C23	121.4 (4)
C12—C11—H11	119.4	C19—C24—H24	119.3
C11—C12—H12	120.3	C23—C24—H24	119.3
			
F1—C4—C5—C6	179.9 (4)	C9—C10—C15—C14	−178.3 (4)
F2—C22—C23—C24	−179.0 (4)	C9—C10—C15—C16	0.1 (6)
O1—C7—C8—N1	0.0 (5)	C10—C11—C12—C13	−1.1 (6)
O1—C9—C10—C11	−176.3 (3)	C10—C15—C16—O2	61.0 (5)
O1—C9—C10—C15	1.6 (6)	C10—C15—C16—N2	−124.5 (5)
O2—C18—C19—C20	−177.2 (4)	C11—C10—C15—C14	−0.5 (6)
O2—C18—C19—C24	1.8 (6)	C11—C10—C15—C16	177.9 (4)
N1—C9—C10—C11	1.7 (6)	C11—C12—C13—C14	1.1 (7)
N1—C9—C10—C15	179.6 (4)	C12—C13—C14—C15	−0.8 (7)
N2—C17—C18—O2	−0.5 (5)	C13—C14—C15—C10	0.5 (6)
N2—C17—C18—C19	179.7 (4)	C13—C14—C15—C16	−178.1 (4)
C1—C2—C3—C4	−1.5 (7)	C14—C15—C16—O2	−120.5 (4)
C1—C7—C8—N1	−175.8 (4)	C14—C15—C16—N2	54.0 (6)
C2—C1—C6—C5	0.7 (7)	C15—C10—C11—C12	0.8 (6)
C2—C1—C7—O1	−12.4 (6)	C16—O2—C18—C17	−0.3 (4)
C2—C1—C7—C8	163.0 (5)	C16—O2—C18—C19	179.5 (3)
C2—C3—C4—F1	−179.1 (4)	C16—N2—C17—C18	1.2 (5)
C2—C3—C4—C5	2.2 (8)	C17—N2—C16—O2	−1.4 (5)
C3—C4—C5—C6	−1.4 (9)	C17—N2—C16—C15	−176.2 (4)
C4—C5—C6—C1	−0.1 (7)	C17—C18—C19—C20	2.4 (8)
C6—C1—C2—C3	0.1 (7)	C17—C18—C19—C24	−178.5 (5)
C6—C1—C7—O1	171.3 (4)	C18—O2—C16—N2	1.1 (4)
C6—C1—C7—C8	−13.3 (7)	C18—O2—C16—C15	176.2 (3)
C7—O1—C9—N1	1.9 (4)	C18—C19—C20—C21	−179.6 (4)
C7—O1—C9—C10	−179.9 (3)	C18—C19—C24—C23	179.2 (4)
C7—C1—C2—C3	−176.2 (4)	C19—C20—C21—C22	0.2 (8)
C7—C1—C6—C5	177.1 (4)	C20—C19—C24—C23	−1.7 (6)
C8—N1—C9—O1	−1.9 (5)	C20—C21—C22—F2	178.6 (4)
C8—N1—C9—C10	−179.9 (4)	C20—C21—C22—C23	−1.4 (8)
C9—O1—C7—C1	175.5 (3)	C21—C22—C23—C24	1.0 (7)
C9—O1—C7—C8	−1.0 (4)	C22—C23—C24—C19	0.6 (6)
C9—N1—C8—C7	1.1 (5)	C24—C19—C20—C21	1.3 (7)
C9—C10—C11—C12	178.8 (4)		

### 1,2-Bis[5-(4-fluorophenyl)-1,3-oxazol-2-yl]benzene Hydrogen-bond geometry (Å, º)

**Table:**

*D*—H···*A*	*D*—H	H···*A*	*D*···*A*	*D*—H···*A*
C5—H5···F2^i^	0.93	2.70	3.470 (5)	141
C8—H8···N2^ii^	0.93	2.56	3.489 (6)	176
C23—H23···F1^iii^	0.93	2.82	3.559 (5)	137
C24—H24···N1^iv^	0.93	2.54	3.449 (5)	168

Symmetry codes: (i) −*x*, −*y*+1, −*z*; (ii) −*x*+1, *y*−1/2, −*z*+1/2; (iii) *x*, *y*, *z*+1; (iv) −*x*+1, −*y*+1, −*z*+1.
